# Angiogenic biomarkers in children with congenital heart disease: possible implications

**DOI:** 10.1186/1824-7288-36-32

**Published:** 2010-04-20

**Authors:** Nagla T El-Melegy, Nagwa A Mohamed

**Affiliations:** 1 Medical Biochemistry Department, Faculty of Medicine, Assiut University, Assiut, (71515), Egypt; 2 Department of Pediatrics, Faculty of Medicine, Assiut University, Assiut, (71515), Egypt

## Abstract

**Background:**

Vascular endothelial growth factor (VEGF), platelet derived endothelial cell growth factor/thymidine phosphorylase (PD-ECGF/TP) and leptin are known as potent angiogenic factors The objective of the study was to evaluate these angiogenic factors VEGF, PD-ECGF/TP and leptin in children with congenital heart disease (CHD) and the factors that lead to angiogenesis in such cases.

**Methods:**

Sixty CHD children were studied and divided into two groups (n = 30); cyanotic-CHD (C-CHD) and acyanotic-CHD (A-CHD). Twenty five healthy children were included as controls.

**Results:**

Significantly higher serum levels of VEGF, PD-ECGF/TP activity and leptin were detected in patients with CHD, particularly in patients with C-CHD. CHD patients with SpO_2 _<90%, pulmonary hypertension (PH), severe pulmonary stenosis (PS), detectable collaterals, cardiomegaly and/or heart failure showed significantly higher levels of these factors than those with higher SpO_2 _or those without these findings.

**Conclusion:**

Hypoxia, PH and PS are important factors that lead to harmful angiogenesis. However, angiogenesis could be essential in some cases of CHD as coarctation of aorta to enhance renal perfusion. This may provide new ways for therapeutic strategies aiming at reducing or promoting angiogenesis in CHD to improve patient's outcome.

## Background

Angiogenesis or neovascularization by capillary sprouting from preexisting vessels is a major physiological event that occurs for example in embryogenesis, wound healing and menstrual cycle. It is also, implicated in certain pathological conditions such as atherosclerosis, diabetic retinopathy, tumor growth, psoriasis and myocardial ischemia. This could occur through the interplay of the endothelial cells, angiogenic mediators, cytokines, growth factors, and adhesion molecules [1].

Children with CHD may experience the development of abnormal vascular channels that become a source of significant morbidity and mortality. However, no entity responsible for these abnormalities has been identified yet [2]. Therefore, such vessels are needed to be closed either before or after the cardiac operation [3]. On the other hand, cases with CHD as coarctation of aorta, enlargement of preexisting or formation of new collaterals is very characteristic and essential for enhancement of renal perfusion as hypertension is not due to mechanical obstruction alone, but almost certainly involves renal mechanisms [4].

Angiogenic growth factors are so called because of their varying ability to induce the proliferation of various cells in vitro, which contribute to the process of angiogenesis in vivo [5]. Vascular endothelial growth factor (VEGF), platelet derived endothelial cell growth factor/thymidine phosphorylase (PD-ECGF/TP) and leptin are known as potent angiogenic factors [5-7]. VEGF is a basic 45 kDa disulfide linked dimeric glycoprotein that binds heparin and is structurally related to platelet derived growth factors. The whole VEGF family consists of at least five members (VEGF A-B-C-D-E) whose effects are mediated via three VEGF receptors (VEGF R-1, VEGF R-2 and VEGF R-3). Through alternate exon splicing of VEGF gene, different mRNA are encoded producing five biologically active proteins (VEGF_121_, VEGF_145_, VEGF_165_, VEGF_189 _and VEGF_206_) [5]. PD-ECGF/TP is identical to TP (EC-2-4-2-4). PD-ECGF/TP is a 47 kDa non glycosylated single chain polypeptide that stimulates chemotaxis for endothelial cells in vitro and of angiogenesis in vivo [8]. Despite a wealth of data linking PD-ECGF and angiogenic pathology, the molecular mechanisms underlying this link have thus for remained obscure [6]. Leptin, the 16 kDa non-glcosylated polypeptide product of the obese gene, is an adipocyte-derived cytokine that regulates food intake and energy homeostasis. Leptin is also, defined as a potent angiogenic factor [9,10]. Understanding of the role of these angiogenic factors in CHD could be important in medical or surgical management of such cases [4].

Aim of work: The present study was designed to evaluate the angiogenic factors (VEGF, PD-ECGF/TP and leptin) in children with CHD and the factors that lead to angiogenesis in such cases.

## Methods

The study included 60 children with CHD diagnosed clinically and by echocardiography. They were attending Cardiology Unite at Children University Hospital, Assiut. They were divided into two groups; C-CHD (n = 30) (16 boys and 14 girls). Their mean age was 3.38 ± 0.78 years and mean weight 10.2 ± 3.2 Kg. A-CHD group (n = 30) (22 boys and 8 girls). Their mean age was 3.20 ± 2.12 years and mean weight 11.46 ± 4.82 Kg. The diseases of C-CHD were 14 Tetralogy of Fallot (TOF), 9 transposition of great arteries (TGA), 7 double outlet right ventricle (DORV). The A-CHD had 11 ventricular septal defect (VSD), 8 pulmonary stenosis (PS), 5 coarctation of aorta, 3 patent ductus arteriosus (PDA) and 3 atrial septa defect (ASD). A control group of 25 apparently healthy children (15 boys and 10 girls), with mean age 3.48 ± 2.31 years and mean weight 13.5 ± 4.7 Kg were included in the study. All patients were subjected to full history taking, clinical and echocardiographic examination. Diagnostic cardiac catheter as preoperative evaluative procedure was done in 15 cases {10 TOF, 2 coarctation of aorta and 3 VSD with pulmonary hypertension (PH)}. Severe PS was considered when the pressure gradient (PG) between the right ventricle and the pulmonary artery is >60 mmHg [11]. PH is defined as mean pulmonary artery pressure >25 mmHg at rest or >30 mmHg during exercise [11]. Severe coarctation of aorta is diagnosed when the PG between the pre and post the coarctation segment was >60 mmHg [11]. Arterial blood oxygen saturation (SpO_2_) was measured by pulse oxymeter. Patients who were operated either with palliative or complete surgical correction of CHD were excluded from the study as hemodynamics and angiogenesis could be altered postoperatively. According to age, weight, hemoglobin level and SpO_2 _level, CHD patients were divided into {<3 ys (n = 39) and >3 ys (n = 21)}, {<10 kg (n = 34) and >10 Kg (n = 26)}, {<10 g./dl (n = 32) and >10 g./dl (n = 28)}, {<90% (n = 35) and >90% (n = 25)} for comparison. The clinical, laboratory, echocardiographic and catheter criteria of the studied CHD patients were presented in tables 1 and 2. Most of the studied patients will undergo total corrective or palliative surgery according to their conditions and will be evaluated postoperatively by echocardiography.

**Table 1:** Clinical, laboratory, echocardiographic and catheter criteria of the studied patients with CHD

Patients groups Studied parameters	C-CHD (n = 30)	A-CHD (n = 30)
Heart failure (n = 18/60)	12	6

Cardiomegaly (n = 25/60)	14	11

SpO_2 _(%)	80.6 ± 7.3	97.1 ± 0.5

Hemoglobin (g./dl)	16.78 ± 2.5	10.0 ± 2.1

PH (n = 12/60)	8	4

Severe PS (n = 21/60)	16	5

collaterals by echo (n = 11/60)	7	4

collaterals by catheter (n = 8/19)	6	2

**Table 2:** Clinical, laboratory, echocardiographic and catheter criteria in different types of CHD

Patients groups			C-CHD (n = 30)			A-CHD (n = 30)				
			
Studied Parameters			TOF (n = 14)	TGA (n = 9)	DORV (n = 7)	VSD (n = 11)	PS (n = 8)	Aortic coarct (n = 5)	PDA (n = 3)	ASD (n = 3)
Heart failure (n = 18/60)			4	5	3	5	-	1	-	-

Cardiomegaly (n = 25/60)			4	4	4	7	-	1	3	2

SpO_2 _(%)			83 ± 3.6	80 ± 2.1	82.6 ± 5.4	97 ± 1.3	96 ± 2.2	96.2 ± 3.1	97.1 ± 4.8	94 ± 3.5

Hemoglobin (g./dl)			15 ± 3.3	16.2 ± 2	16.9 ± 4.3	10.5 ± 2	10 ± 3.2	10.4 ± 3.6	11.9 ± 1.8	10.8 ± 0.9

PH (n = 12/60)			-	5	3	4	-	-	-	-

Severe PS (n = 21/60)			11	2	3	-	5	-	-	-

Collaterals by echo (n = 11/60)			5	1	1	-	2	2	-	-

Collaterals by catheter (n = 8/19)			6	Not done	Not done	-	Not done	2	Not done	Not done

All parents of subjects gave written informed consent prior to the study. The study was approved by the Committee of Ethics in Medical Research at Assiut University, Faculty of Medicine.

Five ml of blood sample were collected by vein puncture under complete aseptic condition. Samples were allowed to clot for 30 minutes and centrifuged at 1000 g for 10 minutes at 4°C. Serum was removed and stored at -20°C in aliquots till time of assay. Serum VEGF levels were measured with Qauantikine VEGF_165 _ELISA kite Catalog number DVEOO (R & D system, Minneapolis, MN) according to method of He et al. [12]. Levels of activity of serum PDECGF/TP were determined by spectrophotometric measurement of the free thymine base produced as a result of the enzyme catalytic reaction [13]. Serum levels of leptin were measured with DRG leptin ELISA Catalog number EIA-2395, supplied by DRG instruments GmbH, Germany [14].

### Statistical analysis

Data were analyzed using statistical package (SPSS Version 11). The results were presented as mean ± SD. Data comparisons were performed using two tailed unpaired student t-test, and Pearson correlation coefficient was used for correlations. A value of p < 0.05 was considered to be significant.

## Results

Significantly higher mean serum levels of the studied bioindices (VEGF, PD-ECGF/TP activity and leptin) were found in patients with CHD in comparison to those of the controls. Patients with C-CHD showed significantly higher levels of the studied bioindices in comparison to those of the A-CHD (P < 0.01 each) {Table 3}. The mean levels of VEGF were significantly higher in C-CHD patients aged more than 3 years than those aged less than 3 years (P < 0.01). Both C-CHD and A-CHD patients weighed more than 10 Kg showed significantly higher mean serum leptin levels than those weighed less than 10 Kg (P < 0.01). Mean serum levels of VEGF and PD-ECGF/TP were significantly higher in C-CHD patients with hemoglobin levels more than 10 g./dl than those of patients with hemoglobin levels less than 10 gm/dl. Significantly higher mean serum levels of the studied bioindices were observed in patients with CHD having SpO_2 _% less than 90% than those with SpO_2 _% more than 90% {Table 4}. CHD patients with PH, those with severe PS and with collaterals showed significantly higher mean serum levels of VEGF, PD-ECGF/TP and leptin than those without PH, with mild PS and without collaterals. Also, significantly higher mean serum levels of VEGF were observed in C-CHD patients with heart failure and those with cardiomegaly in comparison to those without (P < 0.05) {Table 5, 6}. Table 7 shows significant positive correlations in patients with CHD between VEGF and each of PD-ECGF/TP, leptin, age, hemoglobin, pulmonary pressure and PS. and between PD-ECGF/TP and leptin (p < 0.001). Also, significant positive correlations were observed between leptin and each of PD-ECGF/TP and weight. However, a significant negative correlation was observed between VEGF and SpO_2 _%.

**Table 3:** Serum levels of VEGF, PD-ECGF/TP activity and leptin in patients groups and controls

Biomarkers	VEGF (pg/ml)		PD-ECGF (U/ml)		Leptin (ng/ml)	
	
Patients groups	mean ± SD	Range	mean ± SD	Range	mean ± SD	Range
Cyanotic (n = 30) (I)	410.83 ± 102.79	195-600	31 ± 8.22	15-50	41.96 ± 11.05	20-60

Acyanotic (n = 30) (II)	308.66 ± 85.74 a†	180-450	21.16 ± 6.7 a†	12-35	32.93 ± 9.53 a†	15-48

Controls (n = 25) (III)	141.4 ± 55.38 a* b*	65-250	14.96 ± 4.37 a† b‡	9-23	8.88 ± 2.03 a* b*	5-12.5

a: Groups II, III versus I

b: Group III versus II

*: p < 0.001

†: p < 0.01

‡: p < 0.05

**Table 4:** Serum levels of VEGF, PD-ECGF/TP activity and leptin in patients groups in relation to age, weight, hemoglobin and SpO_2 _levels.

Biomarkers			VEGF (pg/ml)		PD-ECGF (U/ml)		Leptin (ng/ml)	
		
Patients groups			C-CHD (n = 30)	A-CHD (n = 30)	C-CHD (n = 30)	A-CHD (n = 30)	C-CHD (n = 30)	A-CHD (n = 30)
	<3 ys (n = 39)		265 ± 100	296 ± 79	29 ± 7	20 ± 10.2	39 ± 12	32 ± 10
Age (years)								
	>3 ys (n = 21)		574 ± 92*	310 ± 91§	34 ± 9.3§	27 ± 6§	43 ± 6.5§	33.6 ± 5.9§

	<10 Kg (n = 34)		400 ± 96	306 ± 74	28.6 ± 7.8	25 ± 4.9	22 ± 4.9	20.9 ± 8.9
Weight (Kg)								
	>10 Kg (n = 26)		413 ± 78§	327 ± 36§	33 ± 6.4§	30 ± 6.7§	43 ± 10†	45.3 ± 9†

	<10 gm/dl (n = 32)		275 ± 102	300.5 ± 90	18.4 ± 6.4	21.2 ± 7.3	41 ± 7.9	31 ± 4.8
Hemoglobin (g./dl)								
	>10 gm/dl (n = 28)		567 ± 95*	313.2 ± 76§	39 ± 9.6†	24.9 ± 6§	43.8 ± 8§	33.7 ± 5.6§

	<90% (n = 35)		582 ± 98	408 ± 59.2	48.3 ± 7.8	33 ± 5.4	49 ± 12	46.8 ± 9.5
SpO_2_ (%)								
	>90% (n = 25)		206 ± 39*	210 ± 65.4*	27 ± 9.6†	18 ± 6.9†	27.8 ± 10.8‡	22 ± 6.2 ‡

*: p < 0.001

†: p < 0.01

‡: p < 0.05

§: not significant

**Table 5:** Serum levels of VEGF, PD-ECGF/TP activity and leptin in patients groups in relation to PH, severity of PS, presence or absence of heart failure and cardiomegaly

Biomarkers					VEGF (pg/ml)		PD-ECGF (U/ml)		Leptin (ng/ml)	
		
Studied parameters					Cyanotic (n = 30)	Acyanotic (n = 30)	Cyanotic (n = 30)	Acyanotic (n = 30)	Cyanotic (n = 30)	Acyanotic (n = 30)
	With (n = 12)				589 ± 89	395 ± 78	39.6 ± 4.4	30 ± 6.0	59.5 ± 6.0	44.6 ± 6.1
**PH** (n = 12/60)										
	Without (n = 48)				276 ± 92*	187 ± 85*	22.2 ± 7.3†	19.9 ± 5.7†	30.2 ± 11†	23.8 ± 5.1†

	Severe (n = 21)				478 ± 74	384 ± 62	47.3 ± 6.6	33 ± 8.9	53.6 ± 8.4	41 ± 6.5
**PS** (n = 24/60)										
	mild (n = 3)				199 ± 52*	220 ± 77†	27 ± 8.9†	17.9 ± 7.4†	30 ± 13.3†	22.8 ± 7.9†

	With (n = 18)				519 ± 79	3854 ± 88	29 ± 6.4	32.2 ± 5.8	52 ± 7.4	41 ± 2.9
**Heart Failure** (n = 18/60)										
	Without (n = 42)				476 ± 68‡	87 ± 74§	22 ± 7.0§	29 ± 8.5§	44 ± 10.6§	33 ± 9.5§

	With (n = 25)				379 ± 55	292 ± 67	39 ± 4.0	32.3 ± 6.0	43 ± 9.9	43 ± 8.2
**Cardiomegaly** (n = 25/60)										
	Without (n = 35)				294 ± 95‡	201 ± 77§	27 ± 6.1§	30 ± 4.9§	42 ± 12§	36 ± 7.0§

*: p < 0.001

†: p < 0.01

‡: p < 0.05

§: not significant

**Table 6:** Serum levels of VEGF, PD-ECGF/TP activity and leptin in the studied patients in relation to presence or absence of collaterals detected by echocardiography and/or catheter

Biomarkers		VEGF	PD-ECGF	Leptin
Collaterals		(pg/ml)	(U/ml)	(ng/ml)
	Collaterals (n = 11)	490.3 ± 77	43.2 ± 7.8	48.6 ± 8.7
Echo (n = 60)				
	No (n = 49)	232.7 ± 80.2*	21.0 ± 9.4†	29.0 ± 8.8†

	Collaterals (n = 8)	512.0 ± 80.3	46.1 ± 9.3	51.5 ± 7.32
Catheter (n = 19)				
	No (n = 11)	266.0 ± 92.0*	27.0 ± 10.5†	26.4 ± 9.9†

*: p < 0.001

†: p < 0.01

**Table 7:** Correlation coefficient (r) between the studied parameters in patients with CHD

Studied parameters	VEGF (pg/ml)	PD-ECGF/TP (U/ml)	Leptin (ng/ml)
**PD-ECGF/TP **(U/ml)	r = 0.847*	-	-

**Leptin **(ng/ml)	r = 0.833*	r = 0.769*	-

**Age **(years)	r = 0.562 ‡	r = 0.266§	r = 0.334§

**Weight **(Kg)	r = 0.206§	r = 0.260§	r = 0.448 ‡

**Hemoglobin **(g./dl)	r = 0.799*	r = 0.260§	r = 0.206§

**SpO_2 _(%)**	r = -0.856*	r = 0.320§	r = 0.268§

**Pulmonary pressure **(mmHg)	r = 0.799*	r = 0.260§	r = 0.288§

**Severity of PS **(mmHg)	r = 0.879*	r = 0.433 ‡	r = 0.527 ‡

**Severity of aortic coarctation **(mmHg)	r = 0.904*	r = 0.662 †	r = 0.531 ‡

**Heart failure**	r = 0.266§	r = 0.280§	r = 0.302§

**Cardiomegaly**	r = 0.205§	r = 0.240§	r = 0.300§

*: p < 0.001

†: p < 0.01

‡: p < 0.05

§: not significant

## Discussion

Abnormal blood vessel proliferation often occurs in patients with CHD and this may represent a form of anomalous angiogenesis, especially in the lungs or myocardium [15].

The present study demonstrated significant higher mean serum levels of VEGF, PD-ECGF/TP activity and leptin in children with CHD particularly the cyanotic group (Table 3).

Angiogenesis normally occurs at variable rates in different organs, because it depends on the tissue regeneration. That response is amplified under conditions that results in cellular hypoxia [2]. Hypoxia (decreased SpO_2_) that leads to polycythemia with increased hemoglobin level is a strong stimulus for angiogenesis. Hu et al. [15] stated that preoperative serum VEGF level elevation was because of hypoxia in the C-CHD, but then became lower after surgery because of the elimination of hypoxia. In contrast, the serum VEGF level significantly increased postoperatively in patients with A-CHD, possibly from myocardial ischemia during cardiopulmonary bypass. Nevertheless, in the C-CHD hypoxia is still the main cause of the high serum VEGF levels rather than myocardial ischemia. The response to hypoxia is primarily mediated by the transcription factor hypoxia-inducible factor-1 (HIF-1) which leads to the induction of a variety of adaptive gene products including VEGF [16]. Further mechanisms leading to hypoxia-induced increase of VEGF production may be related to the often associated features of hypoxia such as tissue damage, necrosis and apoptosis. These events may therefore trigger the release of cytokines and other chemical mediators from the cells of the surrounding tissue, initiating a cascade of events leading to the production of VEGF [5]. These findings could explain the observations of the present study that significant higher mean serum levels of VEGF were detected in C-CHD patients with higher hemoglobin level >10 g./dl than those with lower hemoglobin <10 g./dl and in C-CHD and A-CHD patients with lower SpO_2 _level <90% than those with higher SpO_2 _level >90% (Table 4) and the significant positive correlation between the levels of VEGF and hemoglobin and significant negative correlation between VEGF and SpO_2 _(Table 7). These findings are in agreement with those of Himeno et al. [3] and Suda et al. [17]. However, Ootaki et al [2] found no significant correlation between VEGF level and SpO_2 _or RBCs. They attributed this to the fact that VEGF is classified into subgroups and some types of VEGF are not affected by hypoxia (VEGF-B and C).

In the present study, C-CHD patients with older age >3 ys showed a significantly higher levels of VEGF than younger age <3 ys (Table 4). There were significant positive correlations between VEGF and age (Table 7). These findings are in accordance with those of Himeno et al. [3] who reported that VEGF showed significant age dependence. Hu et al. [15] stated that the myocardial microvessel formation increased with age and this could be attributed to the older the age the longer the duration of angiogenesis formation in C-CHD. Accordingly, they suggested that early surgical intervention may improve the outcome of CHD. However, the study of Rivard et al. [18] reported that the expression of VEGF was impaired in old animals compared with younger ones.

C-CHD patients with heart failure and those with cardiomegaly showed significant higher mean serum levels of VEGF than patients without (Table 5). Early PH in heart failure is characterized by altered vasoreactivity with overexpression of VEGF [19]. Izumiya et al. [20] reported that VEGF is required to maintain myocardial capillary density and that reduction in vascular bed is associated with the transition from compensated hypertrophy to heart failure. Moreover, Tirziu et al. [21] stated that the enhancement of angiogenesis in failing heart is to improve myocardial function. They reported that an increase in size of vascular bed in normal heart leads to increased cardiac mass and hypertrophy paralleled by increased cardiac performance.

It has been shown that TP activity is critical for the angiogenic activity of PD-ECGF/TP. TP hydrolyzes thymidine to 2-deoxy-D-ribose-1-phosphate (2-DDR1P) and thymine. In turn 2 DDR1P is dephosphorylated to 2-deoxy-D-ribose (2-DDR). These ribose sugar metabolites are strong reducing sugars that generate oxygen radical species during the early stages of protein glycation. The induced oxidative stress has been implicated in the release of angiogenic factor (ex. VEGF) [6,22]. In addition, 2-DDR could possibly exert a chemokinetic effect by integrating into the metabolic machinery of endothelial cell generating energy that can serve towards endothelial cell migration which has been suggested to be essential for angiogenesis [22]. Characterizing the angiogenic pathways downstream of TP activity will allow us to uncouple angiogenesis from thymidine phosphorylysis. This is of clinical relevance to block TP-induced angiogenesis downstream of TP activity which could prevent the induction of blood vessel growth [6].

The present study revealed that mean serum activity levels of PD-ECGF/TP activity were significantly higher in CHD patients with low SpO_2 _(Table 4). This finding could be attributed to hypoxia. Griffiths and Dachs [23] reported that the presence of PD-ECGF/TP is a common feature in all diseases characterized by hypoxia and aberrant angiogenesis. They added that the mechanism of hypoxic induction of PD-ECGF/TP may be similar to that of VEGF[22,23]. These findings could explain the significant positive correlation between VEGF and PD-ECGF/TP observed in CHD patients in the present study (Table 7).

The angiogenic function of leptin demonstrates that this endocrine hormone acts as a paracrine growth factor for the vascular system [24]. In addition, leptin also, acts as an indirect angiogenic factor. Interestingly, leptin modulates VEGF-induced vascular activity by synergistically promoting neovascularization in vivo [24]. The study of suganami et al. [7] reported that leptin stimulates neovascularization possibly through the upregulation of VEGF in retinal endothelial cells. This synergism is by upregulation of VEGF mRNA expression via activation of Janus-tyrosine kinase-signal transducer and activator of transcripition (Jak-STAT) [10]. These findings could explain the observed significant positive correlation between VEGF and leptin in the studied CHD patients (Table 7). Hypoxia which is a well-known stimulator of leptin synthesis and secretion [25], it markedly enhances the expression of leptin and VEGF as well as stimulating HIF-1α which transactivate the leptin gene promoter [9]. This could explain the present finding that significant higher serum leptin levels were observed in CHD patients with lower SpO_2 _(Table 4). In the present study, significant higher serum leptin levels were observed in CHD patients with body weight >10 kg than those patients <10 kg (Table 4). Also, significant positive correlation was observed between leptin and body weight (Table 7). Numerous studies have revealed that leptin concentrations are significantly elevated in obese subjects in proportion to the degree of adiposity. The mechanism by which the increase in body fat is translated into increase in serum leptin appears to involve induction of Ob gene expression [14]. However, the study of Hallioglu et al. [26] reported that the leptin regulating axis is intact in cyanotic patients and does not contribute to cachexia of CHD.

Significant higher serum levels of VEGF, PD-ECGF/TP activity and leptin were observed in CHD patients with PH than patients without (Table 5). Also, there was a significant positive correlation between VEGF and level of pulmonary pressure (Table 7). This is in agreement with that of Lam et al. [27] who detected increased expression of VEGF in the pulmonary artery of rats with high pulmonary flow. Mata-Greenwood et al. [28] suggested that increased pulmonary blood flow upregulates expression of VEGF and its receptors, and this may be important in development of the vascular remodeling in the pulmonary artery exposed to high flow and pressure. The characteristic vascular changes include intimal hyperplasia/fibrosis, medial hypertrophy, extensive extracellular matrix modulation, and, in more severe cases, the formation of plexiform lesion [29]. These changes lead to decreased compliance of pulmonary vasculature and changes in vasoreactivity of pulmonary arteries [30].

Significant higher mean serum levels of VEGF, PD-ECGF/TP activity and leptin were observed in CHD with collaterals than those without (Table 6). These finding agree with those of previous studies [2,15,31]. Starnes et al. [31] reported that VEGF appears to be systemically elevated in patients with chronic cyanosis, and may contribute to the formation of extensive collateral vessels. Ootaki et al. [2] reported that, even without major abnormal vessels, patients with C-CHD frequently have high amounts of collateral flow by numerous small collaterals. These secondarily developed systemic pulmonary collaterals might relate to VEGF levels. These findings may suggest that the use of gene therapy of VEGF inhibitors might represent a way to limit collateral development [3]. Also, significant higher serum levels of VEGF, PD-ECGF/TP activity and leptin were observed in CHD patients with severe PS than patients with mild PS (Table 5). Aortopulmonary collateral arteries associated with pulmonary atresia or severe PS may lead to several problems, including significant left to right shunting and pulmonary "steal" from systemic flow during cardiopulmonary bypass. On the other hand, the maintenance of collaterals that extend in cases of severe coarctation of aorta to supply the post stenotic ischemic tissues especially kidneys is very essential for their perfusion. Higher blood pressure in the capillaries of the aorta proximal to the area of obstruction leads to remodeling of the thoracic arteries and of collateral vessels. Promoting angiogenesis in such conditions could be very beneficial to provide better blood supply and to reverse ischemia [4].

## Study limitations

The present study has its own limitations: the broad variation in circulatory dynamics within the studied groups with various CHD. Variability of the underlying hemodynamics and anatomy may make consistent analysis difficult. Another limitation was small number of the studied CHD patients.

## Conclusions

The present study represents an attempt to identify factors that may have an impact on CHD. Hypoxia, PH and PS are important factors that lead harmful angiogenesis and many surgical hazards. On the other hand, angiogenesis could be essential in cases of CHD associated with peripheral ischemia as in coarctaion of aorta to enhance renal perfusion. These findings may aid in understanding of the control mechanisms of angiogenesis in these patients. This may provide new ways of research for therapeutic strategies aiming at reducing or promoting angiogenesis in CHD in order to improve patient's outcome.

## Abbreviations

A-CHD: acyanotic congenital heart disease; ASD: atrial septa defect; C-CHD: cyanotic congenital heart disease; DORV: double outlet right ventricle; PD-ECGF: platelet derived endothelial growth factor; PH: pulmonary hypertension; SpO_2_: arterial blood oxygen saturation; PS: pulmonary stenosis; TGA: transposition of great arteries; TOF: Tetralogy of Fallot; VEGF: vascular endothelial growth factor; VSD: ventricular septal defect.

## Competing interests

The authors declare that they have no competing interests.

## Authors' contributions

Both authors participated in its design, coordination and drafted the manuscript.

NAM collected demographical and clinical data of the studied children. NTE performed the laboratory investigations. All authors read and approved the final manuscript.
